# Effects of mitochondrial dysfunction on the immunological properties of microglia

**DOI:** 10.1186/1742-2094-7-45

**Published:** 2010-08-11

**Authors:** Annette I Ferger, Loretta Campanelli, Valentina Reimer, Katharina N Muth, Irma Merdian, Albert C Ludolph, Anke Witting

**Affiliations:** 1 Department of Neurology, Ulm University, Ulm, Germany

## Abstract

**Background:**

Neurodegenerative diseases are characterized by both mitochondrial dysfunction and activation of microglia, the macrophages of the brain. Here, we investigate the effects of mitochondrial dysfunction on the activation profile of microglial cells.

**Methods:**

We incubated primary mouse microglia with the mitochondrial toxins 3-nitropropionic acid (3-NP) or rotenone. These mitochondrial toxins are known to induce neurodegeneration in humans and in experimental animals. We characterized lipopolysaccharide- (LPS-) induced microglial activation and the alternative, interleukin-4- (IL-4-) induced microglial activation in these mitochondrial toxin-treated microglial cells.

**Results:**

We found that, while mitochondrial toxins did not affect LPS-induced activation, as measured by release of tumor necrosis factor α (TNF-α), interleukin-6 (IL-6) and interleukin-1β (IL-1β), they did inhibit part of the IL-4-induced alternative activation, as measured by arginase activity and expression, induction of insulin-like growth factor 1 (IGF-1) and the counteraction of the LPS induced cytokine release.

**Conclusions:**

Mitochondrial dysfunction in microglial cells inhibits part of the IL-4-induced alternative response. Because this alternative activation is considered to be associated with wound healing and an attenuation of inflammation, mitochondrial dysfunction in microglial cells might contribute to the detrimental effects of neuroinflammation seen in neurodegenerative diseases.

## Background

Activation of microglial cells, the macrophages of the brain, is a common and early hallmark of neurodegenerative diseases and contributes directly to neuronal pathology in virtually all CNS diseases [1]. Activated microglia release a combination of bioactive agents including interleukin-6 (IL-6), tumor necrosis factor alpha (TNFα), and insulin-like growth factor 1 (IGF-1). These bioactive agents have both protective and detrimental consequences for the surrounding brain tissue [2,3]. In most neurodegenerative diseases, levels of pro-inflammatory cytokines such as TNFα increase, suggesting that a pro-inflammatory activation of microglia, as also seen with lipopolysaccharide (LPS) stimulation, outweighs the protective effects of microglia in these diseases. In contrast to pro-inflammatory activation, alternative activation is involved in wound healing and an attenuation of inflammation associated with protective effects. Alternative activation is induced by the type 2 helper T cell cytokine IL-4, which is produced in the CNS and appears to be a critical regulator of neuroinflammation [4]. This alternative activation of microglia is characterized by an increase in arginase activity [5].

The activation of microglia also depends on intrinsic factors. In the SOD1 transgenic mouse model of amyotrophic lateral sclerosis (ALS) the exclusion of mutant SOD1 expression in microglial cells is associated with decreased inflammation and an extension of life span [6]. Expression of mutant SOD1 by microglial cells therefore increases the detrimental effects of these cells. In fact, microglial cells constitutively express not only SOD1 but also other proteins that harbor neurodegeneration-causing mutations, e.g huntingtin and alpha-synuclein, and this might also modulate microglial activation [6-11].

Another hallmark of neurodegenerative diseases are mitochondrial dysfunctions [12]. Neurodegenerative disease-causing mutations like mutated huntingtin have been linked to mitochondrial dysfunction [13], and an impairment of mitochondrial activity has been observed in cells and tissues isolated from patients with neurodegenerative diseases. Furthermore, toxins that inhibit the mitochondrial electron transport chain induce neurodegenerative diseases in humans and animals. For instance, inhibition of complex I (NADH dehydrogenase) with rotenone induces a Parkinson disease-like phenotype in animals with corresponding degeneration of dopaminergic neurons in the substantia nigra of the brain stem. Inhibition of complex II (succinate dehydrogenase) with 3-nitropropionic acid (3-NP) induces a Huntington disease-like phenotype in animals with corresponding degeneration of medium spiny neurons in the striatum [14]. In the 3-NP-induced neuronal degeneration model, and in aged mice, activated microglial cells show signs of mitochondrial dysfunction [15,16]. It is therefore likely that the molecular pathways activated by neurodegenerative disease-causing mutations or toxins that lead to mitochondrial dysfunction are also present in microglial cells. Here we hypothesize that mitochondrial dysfunction will change the immunological profile of microglia. To test our hypothesis we inhibited mitochondrial complexes of the electron transport chain of primary mouse microglial cells and investigated the inflammatory responses of these cells.

## Methods

### Cell culture

Mouse microglial cells in primary cultures were prepared as described previously [17]. Briefly, 1-5 day old C57Bl/6 mice were decapitated according to the guidelines of the animal research center of Ulm University, Ulm, Germany. Meninges were removed from the brains. Neopallia were dissected and enzymatically (1% trypsin, Invitrogen, 0.05% DNAse, Worthington, 2 min) and mechanically dissociated. The resulting cells were centrifuged (200 × g, 10 min), suspended in culture medium (DMEM, Invitrogen) supplemented with penicillin (100 U/ml), streptomycin (100 μg/ml) (Invitrogen) and heat-inactivated fetal bovine serum (10% FBS, PAA); and plated into 75-cm^2 ^flasks (BD Falcon) pre-coated with 1 μg/ml poly-L-Ornithin (Sigma). Cells from the neopallia of two brains were plated into 10 ml per flask. After three days, adherent cells were washed three times with DPBS (Invitrogen) and incubated with serum-supplemented culture media. After 7-14 days in culture, floating and loosely attached microglial cells were manually shaken off, centrifuged (200 × g, 10 min) and seeded into 96-well plates or 6-well plates (PRIMARIA, BD Falkon) at a density of 4 × 10^4 ^or 6 × 10^5 ^cells/well respectively or onto coverslips (15 × 10^4 ^cells/coverslip) in DMEM without serum (DMEM, Invitrogen) supplemented with penicillin (100 U/ml), streptomycin (100 μg/ml) (Invitrogen) and Glutamax (Invitrogen). Cells in the flasks were reincubated with serum-supplemented media after the shake-off. Repopulating microglial cells were removed every week for a total of 4 weeks until fewer microglial cells were observed. For astrocyte cultures, attached cells in the flasks were rinsed once with DPBS, detached (0.05% trypsin, 0,5 mM EDTA) and centrifuged (200 × g, 10 min). Cells were plated onto coverslips (1.5 × 10^4 ^cells/coverslip) in DMEM with 5% FBS and supplemented with penicillin, streptomycin and Glutamax. Coverslips were pre-coated with 1 μg/ml poly-L-ornithine.

### Immunocytochemistry

For immunocytochemical characterization of mouse microglia in culture, we used microglial cells 24-48 h after seeding them on polyornithin coated glass cover slips. Astrocytes on cover slips were used 48-72 h after seeding them. Cells were rinsed with PBS, fixed in 4% paraformaldehyde for 10 min, and rinsed three times with PBS. Cells were permeabilized with Triton-X100 (0.1%) for 5 min and incubated overnight with primary antibodies at 4°C in the present of BSA (1%). We used rat-anti-mouse CD11b at 1:50 (Serotec, MCA74) to label microglia and rabbit-anti-GFAP at 1:200 (Abcam ab7779) to label astrocytes. Bound primary antibodies were detected with Alexa Fluor 568-conjugated goat-anti-rat IgG (CD11b) at 1:200 or Alexa Fluor 488-conjugated goat-anti-rabbit IgG (GFAP) at 1:200. Secondary antibodies were incubated for 1 h at room temperature. Stained cells were rinsed with PBS and mounted on slides with Vectashield with DAPI. Labeling was visualized with an Axiovert 135 microscope. Photographs were taken with an Axiocam Color digital camera. To determine the actual quantity of microglial cells in our microglial culture, we carried out three independent immunocytochemical characterizations of these cultures, made three digital images (20× magnification) of random areas of each immunostaining, and manually counted cell nuclei that were stained by DAPI to determine the total cell number present in the selected area. In the same area, we also manually counted cells that were stained by anti-CD11b and anti-GFAP.

### Treatment of cells

Microglial cells in 96-well plates were stimulated with different doses of rotenone (Sigma) or 3-nitropropionic acid (3-NP) (Sigma) in DMEM without serum for the indicated times. Rotenone was first dissolved in DMSO at a concentration of 1 mM. 3-NP was first dissolved in DPBS at a concentration of 50 mM and the pH was adjusted to 7.2. From these solutions further dilutions were made in DMEM without serum. Microglial cells were stimulated with 10 ng/ml IL-4 (R&D) or/and with 1 μg/ml LPS (Sigma).

### LDH and WST-1 assays

The LDH assay and WST-1 assay were performed as described in the manual for the LDH-Cytotoxicity Assay Kit (Bio Vision) and the WST-1 Assay Kit (Quick Cell Proliferation Assay Kit; BioVision). For a positive cell death control, microglia were treated with 1% Triton-X100 (Sigma) for 30 min.

### Arginase activity assay

To measure the arginase activity of microglial cells we used a modified spectrophotometric assay of arginase as described by Han and Viola [18]. Briefly we prepared a 15 mM thioarginine (Cayman) solution and a 1 mM DTNB (Cayman) solution in PBS. We incubated microglial cells in 96-well plates with 0.9 mM thioarginine and 0.1 mM DTNB in DMEM without serum for a few hours and measured the absorbance at 412 nm. We measured an increase in product formation over time in wells containing microglia in contrast to cell-free wells that did not show a product formation. The product formation in untreated microglial cells (basal) was set to 100% for our calculations.

### ELISAS for IL-6, TNF- α, IL-1 β and IGF-1

The amount of IL-6, TNF-α, IL-1 β and IGF-1 was determined with specific ELISAs (IL-6, TNF-α and IL-1β: BioLegend, IGF-1, R&D Systems) as described in the manuals. After incubation with the toxins, IL-4 and/or LPS for the indicated times supernatant was used for the corresponding ELISAs. For the quantification of IL-1 β microglial cells were stimulated with 3 mM ATP (Sigma) for 30 min before the collection of the supernatant. This treatment is known to release the IL-1β from the cells into the supernatant [19]. For the remaining cells in the 96-well plates, protein assays (Bio-RAD Dc Protein Assay) were performed as described in the manual. The concentrations of the cytokines and growth factor were calculated in pg/mg protein.

### Western blot

Microglial cells in six-well plates were incubated and stimulated as described above. Cells were washed with ice cold PBS and lysed with RIPA-Buffer (150 mM NaCl, 10 mM Tris, 0.1% SDS, 1% Triton-X-100, 1% Deoxycholate, 5 mM EDTA, pH 7.4) with protease inhibitors (Roche, 04693159001). Whole cell lysates were electrophoresed (SDS/PAGE), transferred to polyacrylamide gels, and immunoblotted using antibodies directed against arginase 1 (BD Bioscience, 6106708) or tristetraprolin (TTP) (Santa Cruz, G-20: SC-12565). Afterwards the blots were stripped (0.2 M NaOH) and blotted against tubulin (Sigma, T7816) or GAPDH (Santa Cruz, SC-25778). Samples were scanned and analyzed with the Quantity One program (Bio-Rad). TTP and arginase 1 expression was analysed in relation to tubulin- or GAPDH expression

### Statistics

The values presented in the graphs correspond to mean ± SEM of at least three independent experiments. One-way ANOVA with a specified post-test was used to assess differences between groups. Asterisks indicate statistically significant differences with * p < 0.05, ** p < 0.01 and *** p < 0.001.

## Results

### Mitochondrial toxins have no effect on LPS-induced release of pro-inflammatory cytokines

**Figure 1:**
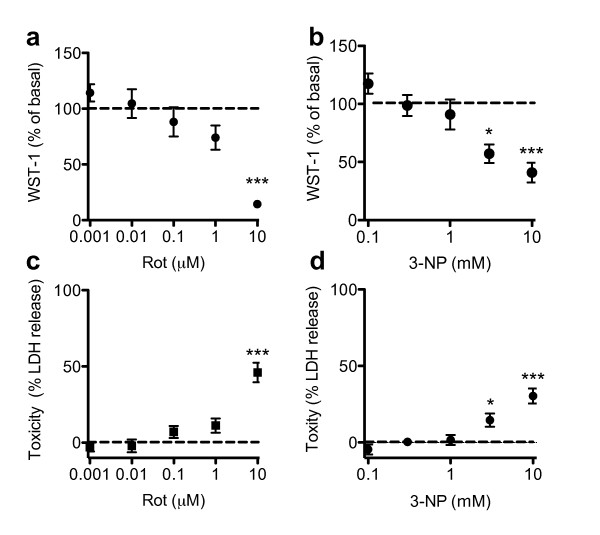
**Mitochondrial toxins induce dose-dependent cell death of microglial cells**. Microglial cells were treated with rotenone or 3-NP. After 24 hrs, cell viability (WST-1) and cell death (LDH) were measured as described in the Methods. Values are mean ± SEM for independent measurements. n = 8-18 wells (i.e., 4-9 separate experiments performed in duplicate); * = p < 0.05, *** = p < 0.001, significantly different from untreated cells (ANOVA followed by Dunnett's Multiple Comparison test).

Mitochondrial toxins like 3-NP and rotenone have been reported to affect cell viability. To exclude toxic effects of these mitochondrial inhibitors on our experimental readouts, we examined the effects of 3-NP and rotenone on cell viability using a WST-1 assay, which measures mitochondrial dehydrogenase activity, and an LDH assay, which measures release of LDH by dead cells. As expected, rotenone and 3-NP both induce a dose-dependent decrease in mitochondrial dehydrogenase activity (Fig 1a, b). A significant reduction of mitochondrial dehydrogenase activity was associated with reduced cell viability as measured by LDH release (Fig 1c, d). The concentrations of 3-NP (0.3 mM and 1 mM) and rotenone (0.02 μM and 0.2 μM) that we used in further experiments did not induce toxic effects (Table 1).

**Table 1:** Rotenone and 3-NP do not affect cell viability

	WST-1 (% of basal)			
	
Condition	Control	LPS	IL-4	LPS + IL-4
Control	100	174 ± 24	121 ± 19	156 ± 24
0.3 mM 3-NP	96 ± 24	158 ± 23	89 ± 24	132 ± 29
1 mM 3-NP	76 ± 25	141 ± 42	107 ± 31	115 ± 29
0.02 μM Rotenone	146 ± 12	194 ± 42	118 ± 21	132 ± 26
0.2 μM Rotenone	115 ± 21	115 ± 38	114 ± 33	111 ± 27

Microglial cells were treated with rotenone or 3-NP and stimulated with 10 ng/ml IL-4. After 24 hrs, 1 μg/ml LPS was added for 18 hrs. The WST-1 assay was performed as described in Methods. Values are mean ± SEM for independent toxicity measurements. n = 6 wells (i.e., 3 separate experiments performed in duplicate); not significantly different from control (ANOVA followed by Dunnett's Multiple Comparison test).

**Figure 2:**
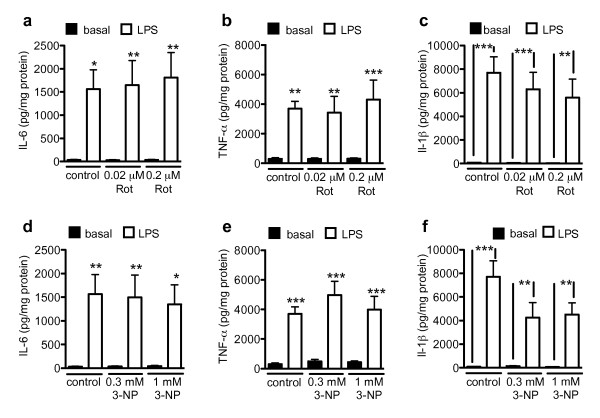
**Mitochondrial toxins do not change LPS-stimulated cytokine production**. Microglial cells were treated with rotenone or 3-NP. After 24 hrs, 1 μg/ml LPS was added for 18 hrs. IL-6 (a, d), TNF-α (b, e) and IL-1 β (c, f) were measured as described in Methods. Values are mean ± SEM for independent cytokine measurements. n = 6-16 wells (i.e., 3-8 separate experiments performed in duplicate); *= p < 0.05, **= p < 0.001, ***= p < 0.001, significantly different from corresponding basal (ANOVA followed by Bonferroni's Multiple Comparison test).

LPS is the prototypical inflammatory activator of microglia in *in vitro *assays. We investigated whether the mitochondrial toxins 3-NP and rotenone, both of which affect the mitochondrial electron transport chain, have an effect on this LPS-induced inflammatory activation of microglial cells. Stimulation of primary mouse microglial cells with 1 μg/ml LPS for 18 h induced production of the pro-inflammatory cytokines IL-6, TNF-α and IL-1β (Fig 2). The mitochondrial toxins 3-NP and rotenone had no significant effect on basal or LPS-stimulated production of IL-6, TNF-α or IL-1β (Fig 2). Thus, LPS-induced inflammatory activation in regard to the production of IL-6, TNF-α or IL-1β seems not to be dependent on the integrity of the mitochondrial electron transport chain.

**Figure 3:**
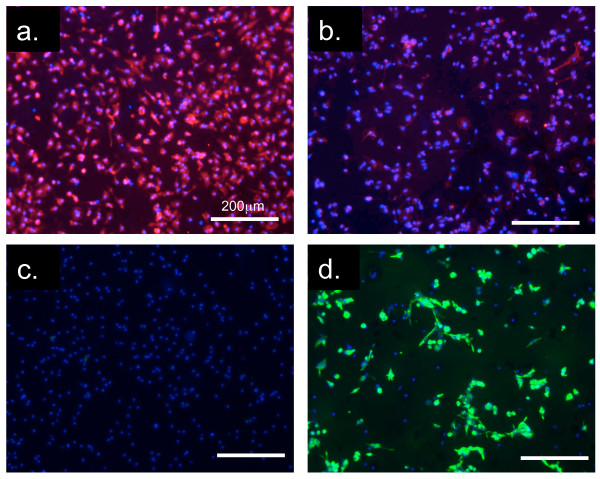
**Immunocytochemical characterisation of cultures of mouse microglia**. Cultures of microglia were immunostained with anti-CD11b (a) and anti-GFAP (c). Cultures of astrocytes were immunostained with anti-CD11b (c) and anti-GFAP (d). All cells were also counterstained with DAPI (blue) to identify the cells' nuclei.

Cultures of primary mouse microglial cells typically contain small amounts of other types of cells, such as astrocytes [20]. We sought to determine the purity of our primary mouse microglial cells that were cultured for 24 h after plating. Using an antibody against CD11b, a protein specific to macrophages and microglia, we determined that 90.3 ± 4.9% of the cells in the cultures were microglia (Fig. 3). An antibody against the astrocyte marker GFAP stained 1 ± 0.9% of the cells (Fig. 3). Therefore, the cultures used in this study were highly enriched in microglial cells.

### Mitochondrial toxins inhibit IL-4-induced arginase activity

**Figure 4:**
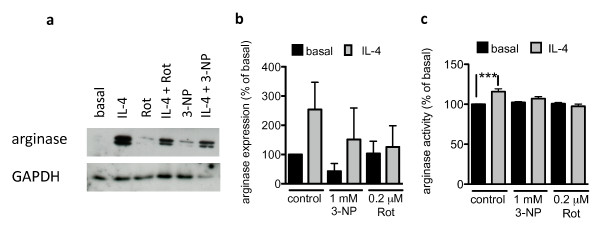
**Mitochondrial toxins inhibit IL-4-induced arginase activity**. Microglial cells were treated with rotenone or 3-NP and stimulated with 10 ng/ml IL-4 for 24 hrs, and arginase expression was analysed by western blot. A representative blot is shown in (a). Quantification of three western blots (b). Microglial cells were treated with rotenone or 3-NP and stimulated with 10 ng/ml IL-4 for 32 hrs. Arginase activity was quantified after 5 h as described in Methods (c). Values are mean ± SEM for independent arginase activity measurements. n = 16-22 wells (i.e., 8-11 separate experiments performed in duplicate); ***, p < 0.001, significantly different from corresponding basal (ANOVA followed by Bonferroni's Multiple Comparison test).

IL-4 induces an alternative activation of macrophages and microglia that is characterized by an increase in arginase activity and expression. This IL-4-induced alternative activation is anti-inflammatory and is involved in wound healing and attenuation of inflammation. To investigate whether mitochondrial toxins have an effect on this anti-inflammatory activation, we incubated primary mouse microglial cells with 10 ng/ml IL-4, with or without the mitochondrial toxins rotenone or 3-NP. IL-4 treatment increased arginase activity and expression (Fig 4). This increase in arginase activity and expression was reduced by 3-NP and rotenone (Fig 4). Thus, IL-4-induced alternative activation might be dependent on a functional mitochondrial electron transport chain.

### Mitochondrial toxins inhibit the IL-4-induced reduction in production of pro-inflammatory cytokines

IL-4-induced, alternatively activated macrophages are known to counteract the production of pro-inflammtory cytokines [21]. We investigated whether mitochondrial toxins changed these IL-4-induced anti-inflammatory effects.

**Figure 5:**
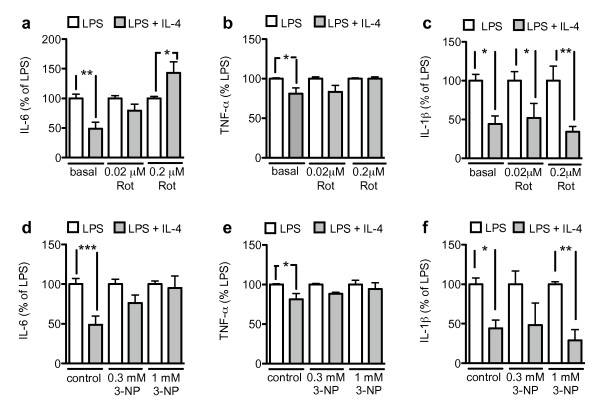
**Mitochondrial toxins inhibit the IL-4-induced decrease in LPS-stimulated IL-6 and TNF-α release**. Microglial cells were treated with rotenone or 3-NP and stimulated with 10 ng/ml IL-4. After 24 hrs, 1 μg/ml LPS was added for 18 hrs. IL-6 (a, d), TNF-α (b, e) and IL-1β (c, f) were measured as described in Methods. Values are mean ± SEM for independent cytokine measurements. n = 6-16 wells (i.e., 3-8 separate experiments performed in duplicate); *= p < 0.05, **= p < 0.001, ***= p < 0.001, significantly different from corresponding basal (ANOVA followed by Bonferroni's Multiple Comparison test).

IL-4-stimulated microglial cells reduced LPS-stimulated IL-6 and TNF-α secretion (Fig 5a, b, d, e). The mitochondrial toxins 3-NP and rotenone inhibited this IL-4-induced reduction of IL-6 and TNF-α release (Fig 5a, b, d, e). IL-4-stimulated microglial cells also reduced LPS-stimulated IL-1β production (Fig 5c, f), but 3-NP and rotenone did not have an effect on this IL-4-induced reduction in IL-1β (Fig 5c, f). This suggests that some responses associated with alternative activation, such as counteraction of the production of IL-6 and TNF-α, depend on fully functional mitochondrial respiration.

### Mitochondrial toxins inhibit IL-4-induced IGF-1 release

**Figure 6:**
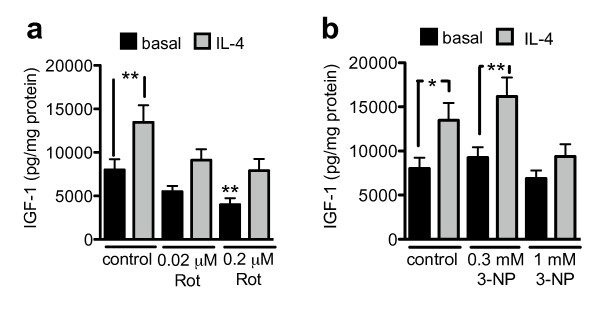
**Mitochondrial toxins inhibit IL-4-induced IGF-1 release**. Microglial cells were treated with rotenone or 3-NP and stimulated with 10 ng/ml IL-4. After 24 hrs, IGF-1 was measured as described in Methods. Values are mean ± SEM for independent IGF-1 measurements. n = 14-16 wells (i.e., 7-8 separate experiments performed in duplicate); *= p < 0.05, **= p < 0.001, ***= p < 0.001, significantly different from corresponding basal (ANOVA followed by Bonferroni's Multiple Comparison test).

IL-4-induced, alternatively activated microglia are known to produce and secrete the neuroprotective insulin-like growth factor-1 (IGF-1) [22,23]. We investigated if this neuroprotective effect of alternative activation is also dependent on the mitochondrial electron transport chain. Microglial cells treated with IL-4 increased IGF-1 release (Fig 6). 3-NP did not have an effect on the IL-4-induced increase in IGF-1 release at 0.3 mM, but attenuated this Il-4-induced response at 1 mM. Rotenone attenuated the IL-4-induced increase in IGF-1 release at all investigated doses, and had a significant inhibitory effect on basal, non-IL-4-induced IGF-1 release at 0.2 μM (Fig 6). Again, the neuroprotective effect associated with IL-4-induced production of IGF-1 is dependent on a functional electron transport chain.

### Mitochondrial toxins inhibit IL-4-induced tristetraprolin expression

**Figure 7:**
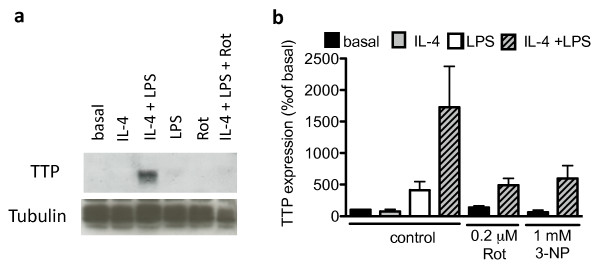
**Mitochondrial toxins inhibit IL-4-induced expression of TTP**. Microglial cells were treated with rotenone and stimulated with 10 ng/ml IL-4. After 4 hrs, 1 μg/ml LPS was added for 18 hrs and TTP expression was analysed by western blot. A representative blot is shown in (a). Quantification of western blots (b). Values are mean ± SEM for three to seven independent western blots.

One mechanism by which IL-4 inhibits the release of pro-inflammatory cytokines is through increased expression of tristetraprolin (TTP) via a mechanism mediated by the transcription factor STAT6 [24]. TTP is an RNA-binding protein that promotes decay of AU-rich element (ARE)-containing mRNA, like TNF-α. We investigated the effect of the mitochondrial toxin rotenone on IL-4-induced expression of TTP. The mitochondrial toxin rotenone inhibited IL-4-induced TTP expression (Fig. 7). Thus a functional change in electron transport chain has an influence on IL-4-induced expression of TTP.

## Discussion

In this study we show that mitochondrial dysfunction in mouse microglial cells inhibits specific responses of alternative activation, whereas classic activation remained unchanged. Evidence that mitochondrial dysfunction inhibits alternative activation is: first, that the mitochondrial toxins 3-NP and rotenone inhibited IL-4-induced arginase activity and expression. The second line of evidence is that these toxins also inhibited IL-4-induced counteraction of the production of the pro-inflammatory cytokines IL-6 and TNF-α, likely by inhibiting IL-4-induced expression of TTP, an RNA-binding protein that promotes decay of AU-rich mRNA. Furthermore, mitochondrial toxins also inhibited IL-4-induced production of the neurotrophic growth factor IGF-1. However mitochondrial dysfunction seemed not to inhibit every aspect of the IL-4 response, as the mitochondrial toxins did not inhibit IL-4-induced counteraction of the production of IL-1β. We conclude, therefore, that some, but not all, of the responses associated with the alternative activation status of microglia are dependent on a functional mitochondrial electron transport chain. In addition we found that classic activation of microglia, which was induced by stimulation with LPS, is not affected by mitochondrial toxins, suggesting that the classic activation might be independent of the mitochondrial function.

In support of our data are findings in peripheral macrophages, where alternative activation is dependent upon functional mitochondria. Inhibition of mitochondrial respiration in macrophages inhibits induction of arginase activity, a hallmark of anti-inflammatory alternative activation [25]. Furthermore inhibition of mitochondrial respiration in macrophages abolishes the anti-inflammatory effects of IL-4 on LPS-induced secretion of IL-6 and TNF-α, whereas this inhibition of mitochondrial respiration does not have an effect on the inflammatory activation of macrophages by LPS [25].

In contrast to Vats et al. [25] we found one IL-4-induced response that is not affected by the mitochondrial toxins used in this study. The IL-4-induced anti-inflammatory effect on LPS-induced secretion of IL1-β by microglial cells [26] was not inhibited by mitochondrial toxins. Our finding suggests that IL-4-induced alternative activation is not completely dependent on mitochondrial respiration. Our findings are in agreement with the notion that mitochondrial inhibitors do not alter IL-4-induced STAT-6 signalling, which is required for the anti-inflammatory response [25]. IL-4 induces the STAT-6-dependent anti-inflammatory response by up-regulation of the mRNA binding protein TTP [24]. TTP promotes decay of AU-rich element- (ARE-) containing mRNA, such as that of TNF-α and IL-6. Mice lacking TTP develop an inflammatory syndrome characterized by arthritis, dermatitis and cachexia as a consequence of enhanced stability of TNF-α mRNA and the resulting excess TNF-α production. We have shown that IL-4 increases LPS-induced TTP expression and that this increase is inhibited by mitochondrial toxins. This represents a possible molecular mechanism for the toxin-induced decrease in IL-4-mediated inhibition of release of TNF-α and IL-6.

Microglial cells treated with rotenone produce superoxide, which leads to significant dopaminergic neuronal cell death [27-29]. It is therefore possible that oxidative stress is involved in mitochondrial toxin-induced inhibition of IL-4-mediated anti-inflammatory effects. TTP phosphorylation and cellular location are influenced by oxidative stress [30]. Interestingly, treatment of cells with the mitochondrial uncoupler FCCP (carbonyl cyanide p-trifluoromethoxyphenyl-hydrazone) induces co-localization of TTP with stress granules, whereas arsenite-induced oxidative stress induces assembly of phospho-TTP:14-3-3 complexes. Both effects are likely associated with inhibition of TTP-promoted decay of mRNA. However, inhibition of TTP may also have an indirect inhibitory effect on inflammatory responses, as HIF-α mRNA and the NF-κB pathway have been shown to be regulated by TTP [31,32].

In contrast to Klintworth et al. [33] and our study, studies by Zhou and colleagues [34] reported a stimulatory effect of rotenone on basal secretion of pro-inflammatory cytokines by primary rat microglia. This discrepancy could be due either to species differences or to an effect of different culture conditions.

Mitochondrial dysfunction in microglial cells have been observed in several animal models of neurodegeneration and aging. In the 3-NP-induced neuronal degeneration model in rats, most mitochondrial dysfunctional signals are associated with activated microglial cells [16]. In aged mice, mitochondrial DNA damage is most pronounced in microglial cells, especially compared to neurons [15]. Hayashi et al. [15] discuss the extremely slow mitochondrial turnover in microglia and the idea that abundant damaged mitochondria may therefore accumulate in microglia. In line with this is the finding that microglial cells in brain are self-maintaining cells that are normally not renewed by bone marrow-derived progenitor cells [35,36], and are therefore prone to an accumulation of mitochondrial damage. Further research is necessary to determine whether mitochondrial dysfunction occurs in microglial cells in other neurological diseases.

## Conclusions

In summary, we have shown that mitochondrial dysfunction in mouse microglial cells inhibit some aspects of alternative activation, whereas classic activation seems to remain unchanged. If, in neurological diseases, microglial cells are also affected by mitochondrial dysfunction, they might not be able to induce a full anti-inflammatory alternative response and thereby exacerbate neuroinflammation. This would be associated with detrimental effects for the CNS since wound healing and attenuation of inflammation would be impaired. Reconstituting the alternative microglial response might therefore be a novel strategy to halt disease progression.

## Competing interests

The authors declare that they have no competing interests.

## Authors' contributions

AW conceived of the project in discussions with ACL. AIF, IM, VR and AW performed the stimulation, the toxicity studies and ELISAs under AW's supervision. AIF performed the arginase activity experiments under AW's supervision. LC performed the TTP expression experiments under AW's supervision. KNM performed the arginase expression experiments under AW's supervision. VR and IM performed the immunocytochemistry experiments under AW's supervision. AIF, VR, LC, KNM and AW performed the statistical analysis. AW prepared the manuscript. All authors have read and approved the final version of this manuscript.
